# Two Cases of Ovariotomy

**Published:** 1876-09

**Authors:** A. Reeves Jackson

**Affiliations:** Surgeon-in-Chief of the Woman’s Hospital of the State of Illinois; Lecturer on Diseases of Women in Rush Medical College, Etc.


### TWO CASES OF OVARIOTOMY.

By A. REEVES JACKSON, A.M., M.D.,

Surgeon-in-Chief of the Woman's Hospital of the State of Illinois; Lecturer on Diseases of Women in Rush Medical College, Etc.

(Read before the Chicago Society of Physicians and Surgeons, June 12, 1876.)

### Case I.—*Polycystic Ovarian Tumor complicated with Ascites — Ovariotomy — Death from Exhaustion.*

Mrs. H., thirty-two years of age, married, and the mother of two children, the youngest twenty-two months old, rode a long distance on a cold day in April, 1872. She took a severe cold and noticed in two or three days an enlargement of the lower part of the abdomen, not greater on one side than on the other. Three months later, Dr. C. T. Cory, of Grant county, Wis., after examining the case, pronounced it one of ovarian dropsy. This opinion was subsequently confirmed by Dr. Darius Mason, of Prairie du Chien. Subsequently, in December, the case came under the care of Dr. J. B. Cory, of Patch Grove, Wis. He found the abdomen excessively distended, and fluctuation evident in every part. There was partial resonance in both flanks and in the epigastric region; dullness everywhere else; change of posture produced no change in this respect. The uterus was slightly prolapsed, otherwise normal. The patient sufferred from dyspnœa, digestion was impaired, and she was greatly emaciated.

In order to give present relief, as well as to complete the diagnosis, the patient was tapped by Dr. Cory, through the abdominal wall, January 2, 1873. He removed fifty pounds of "clear, limpid, amber-colored fluid, free from albumen." After the removal of the fluid Dr. Cory failed to discover any tumor or sac. The patient recovered rapidly. The fluid reaccumulated slowly, but not to such an extent as to occasion distress.

April 1st, following, the patient discovered a tumor in the right iliac region about the size of an orange, hard and moveable. It grew rapidly, so that in two months it was as large as a child's head, and reached almost to the umbilicus; by the last of August it had approached the edge of the ribs. At this time the uterus was drawn up so that the os could be reached only with much difficulty.

She now grew rapidly worse. She had several attacks of peritonitis, suffered greatly from dyspnœa, had a quick pulse and lost flesh.

On the last of January, 1874, her symptoms were so distressing that she was again tapped, at her earnest solicitation. Six pounds of thick gelatinous amber-colored highly-albuminous fluid were removed. The operation was followed by a severe attack of peritonitis, which lasted eighteen days.

I was engaged to perform ovariotomy at the patient's home, and appointed February 25th, as the time. I arrived there very early on the morning of that day, and learned from Dr. Cory that on the evening of the 23d the patient had fallen asleep near an open window, and, that as a result of this act of imprudence she was now suffering from a very severe local peritonitis.

I found her in an exceedingly unfavorable condition. She had a good deal of pain in the left iliac region, attended with nausea and vomiting, tongue and skin dry, pulse 130, temperature 103°. I was very loth to operate under such unpromising circumstances, but as the operation appeared to offer the only possible chance of saving the patient's life, I agreed to submit the decision of the question to the medical gentlemen whom Dr. Cory had invited to be present. These were Drs. Darius Mason, John Conant, T. W. Howes, H. P. Gritner, F. R. Millard, and Thomas Chambers. The attending physician, Dr. J. B. Cory, and Louis Freidericks, medical student, were also present. On consultation with these gentlemen it was decided that the operation should be proceeded with, which was accordingly done.

The patient having been etherized, an incision three inches in length was made in the linea alba and quickly deepened to the peritoneum. When the bleeding ceased, the peritoneum was opened and several pounds of ascitic fluid escaped. The cyst, of a pearl-blue color, then came into view, and a prostatic catheter passed over its surface showed the existence of tolerably dense adhesions on both lateral aspects. These were broken down and a large trocar pushed into the most prominent part of the cyst. Its contents were too viscid and dense, however, to flow through the cannula. The latter was therefore withdrawn, the opening in the cyst wall enlarged and the hand passed into the interior of the growth. The contents of the latter were then scooped out by the hand, the patient being at the same time turned upon her side in order to prevent any of the cystic fluid from entering the abdominal cavity. The tumor was found to be adherent by nearly its entire posterior surface to the intestines and pelvic walls. Many of these were separated by scissors, and in several places portions of the cyst wall were cut out and the inner portion peeled off, leaving the united peritoneal sufaces together. There was a great deal of hemorrhage, and much time was consumed in controlling it. The cyst was found to spring from the right ovary. The left ovary was also diseased and was removed. As it seemed almost inevitable that there would be some oozing of blood from the torn surfaces after the closure of the wound, a drainage tube of india rubber was placed behind the uterus leading from Douglass' cul-de-sac through the vaginal wall into the vagina. The wound was then closed with sutures of silver wire placed half an inch apart, and the dressing completed by adhesive strips, a compress of folded flannel, and a flannel bandage surrounding the body. The weight of the tumor was thirty-two pounds.

The operation lasted two and a half hours, and during its continuance the patient several times became very much depressed, and the pulse quite imperceptible at the wrist. At its conclusion she was placed in bed, had bottles of hot water about her person, and brandy was freely given.

At 2 o'clock P. M., half an hour after she was put to bed, her pulse was 140, small and thready. She did not rally at all. At 7 o'clock on the following morning, after a restless, sleepless night, her pulse was 150, respiration 10, and temperature 105°. She began vomiting a very dark, ropy mucus, and, sinking rapidly, died at 11 o'clock A. M.

CASE II.—*Polycystic Tumor of Right Ovary—Ovariotomy—Symptoms of Septicemia—Recovery retarded by Parotitis and Phlegmasia Dolens.**

H. G., a resident of Aurora, Ill., married, aged forty-nine years, consulted me March 1, 1874, relative to an enlargement of the abdomen, accompanied with great irritability of the bladder and urethra. She gave me the following history: Menstruation commenced at thirteen, the function being irregular down to the age of thirty-four, when she married. It then became regular, and continued so to appear until October last, when, after a few months of irregularity, it ceased altogether. About eighteen months ago she first noticed an enlargement of the abdomen. For the first year the swelling seemed to appear and disappear alternately, but during the past six months it has been permanent, and has steadily increased in size.

On examination, I found the abdomen tense and as large as at full term of pregnancy; there was no fluctuation and no perceptible tumor; the uterus was in a state of extreme retroversion. During the next four months her strength rapidly failed and her size greatly increased. In July I took her to Chicago for consultation with Dr. Jackson. He decided that there was a tumor, probably ovarian, and advised waiting until further development should make operative interference necessary, when ovariotomy should be performed.

She continued to enlarge, and the lower extremities became œdematous; she also complained of pain in the left side, referred to the region of the heart and left lung.

On October 27th, in consequence of her extreme suffering, I removed, by tapping, sixteen pounds of fluid having the color and consistence of new cider. On the withdrawal of the fluid, a tumor extending from the bones of the pelvis on the right side across the abdomen to within two inches of the bones on the left side, was clearly perceptible. Its density varied in different parts, being softest on the left side. After

* The account of this case is furnished by the attending physician, Dr. Jennie G. Brown, of Aurora, Ill.

the operation the uterus resumed its normal position, and the patient was much relieved for several weeks. The fluid re-accumulated, however, and ovariotomy having been decided upon, the operation was performed on December 10th, 1874, by Dr. Jackson, in the presence, and with the assistance of Drs. Jennie G. Brown, J. H. Etheridge, W. C. Lyman, Sibelia F. Baker, Alex. Sterl, R. J. Patterson, Huntley, Brigham, Bartlett, Hawley, Hurlbut and Robbins.

*The Operation.*—The patient having been fully anæsthetized with the bichloride of methylene, administered by Prof. Etheridge, an incision was made in the linea alba, extending from a point about two inches below the umbilicus to one about the same distance from the pubis. This was deepened until the peritoneum was brought into view. All bleeding having ceased, the peritoneum was lifted by a tenaculum and slit upon a director nearly the entire length of the external incision, exposing the wall of the cyst. Adhesions were then searched for by means of a sound swept over the surface of the tumor, and afterwards by the hand previously dipped in artificial serum,* kept at a temperature of 98°. Several were found on the front and lateral aspects of the tumor, and being slight, were broken down by the hand, but some more extensive and of a firmer character, discovered posteriorly, were left to be dealt with subsequently. A trocar was now passed into the upper part of the most prominent cyst, giving exit to a dark amber-colored fluid, closely resembling that which had previously been removed by the tapping. The first cyst having been evacuated, the trocar was re-introduced through the cannula and passed onward, tapping successively others, the contents of each presenting differences in color and consistence. The tumor not being sufficiently reduced in size by this means, the cannula was withdrawn and the opening into the cyst wall enlarged sufficiently to enable the operator to introduce his hand into the interior of the tumor; he then broke down the intervening walls between a number of other

* Made by mixing the whites of two eggs with a tablespoonful of common salt, and adding two quarts of water.

cysts, and permitted their contents to escape. This, too, failed to lessen the size of the growth sufficiently to allow its extraction, and the incision was prolonged to the left of, and about one inch above, the umbilicus. The tumor was carefully brought through the incision, and discovered a number of adhesions attaching it to the intestines and omentum. These were separated by fingers and scissors, and the tumor was finally only held by its pedicle, which was seen to arise from the broad ligament of the right side. The pedicle was temporarily clamped and the cyst cut away; it was subsequently transfixed by a needle in a fixed handle at a point about an inch from its cut extremity, its two halves tied separately with strong silk ligatures, the ends of which were cut off closely, and the pedicle dropped back. The left ovary was found to be diseased, and adherent to the cyst. It was removed. The bleeding points of the divided adhesions were secured with fine silk ligatures, five in number, their ends cut short.

The appearance and condition of the uterus were very peculiar. It was of normal size and its fundus was studded with a number of subperitoneal growths varying in size from a grape-seed to that of a large currant, rough and unsymmetrical, and as hard as stone. They seemed to be small fibroids which had undergone calcaeous degeneration. They were not disturbed.

A drainage tube of india rubber was passed from Douglas' cul-de-sac through the posterior vaginal wall, and left *in situ*. The abdominal cavity was carefully cleansed of blood and cystic fluid by repeated washings of warm carbolized water, and sponged until quite dry. A flannel compress, wrung out of warm water, was placed over the intestines while the needles were passing through the lips of the wound, which was closed with nine silk sutures. The dressing was completed by broad strips of adhesive plaster, over which was placed successively a compress wet with carbolized water, and a layer of cotton batting, the whole being finally surrounded with a flannel bandage.

The patient was then placed in a well-warmed bed, with hot water bags near her feet, and lightly covered with blankets. She soon reacted from the effects of the operation and the anæsthetic. The latter acted admirably. The patient was under its full influence two hours and twenty-six minutes. She had no vomiting during or after the operation, although for several days there was much nausea.

*Progress.*—Dec. 8, P. M. Pulse 88, skin moist, temperature 98°, comfortable.

12th, 10 A. M. During the past two days the pulse has steadily increased to 130, temperature remains at 98°. The skin has been moist, urine abundant, and the patient has had but little pain. Bloody fluid has steadily passed through the drain tube. She is now restless, weary, and has a good deal of nausea; seems better when deprived of all fluids. She has had since the operation enough opium, either by the mouth or rectum, to control pain and permit sleep.

13th, 3 P. M. Has been troubled to-day with eructation of gas; has less nausea, however. Pulse 120, temperature 98°, skin hot and dry, head feels light and dizzy.

14th, 5 P. M. Dr. Jackson came and removed the sutures this morning. The wound was found entirely closed. There has been no distension of the bowels, and gas passes freely *per rectum.* Patient complains of weariness and numbness. Pulse varied during the day from 108 to 120; temperature 98°.

15th, 10 A. M. Pulse 128, temperature 97°. Face flushed, head dizzy, sweet taste in the mouth and sweetish odor of breath; sleep disturbed with unpleasant dreams, and mind dull and cloudy on waking. The drain tube not seeming to act well, I removed it and found the upper portion filled with bloody pus; no fluid followed its withdrawal.

16th. Pulse 128, temperature 96°. Dr. Jackson came to-day and found a hard mass occupying Douglas' space. He passed a curved trocar and cannula into it, and gave exit to one pint of dark bloody offensive fluid, subsequently washing out the part through the cannula with warm carbolized water. This procedure was followed by improvement, the patient becoming at once bright and cheerful. The temperature sank to 94°, and the patient began to perspire freely.

18th. The septicæmic symptoms and the pelvic hardness returning, Dr. Jackson was again sent for, and last night again drew off about a pint of the same offensive bloody fluid as before, and this time left the cannula *in situ*. The patient seemed greatly relieved; but this evening the colon is much distended with gas. Pulse 116, temperature 96°.

19th. Pulse 100, temperature 96°, skin moist, appetite good. Bloody fluid, with fibrinous flakes, pass through the cannula, and two or three times daily the pelvic and abdominal cavities are washed out through the tube. The tympanitic distension is relieved by the use of an enema.

25th. Progress has been steady during the past five days. The pulse has varied from 88 to 100, and the temperature has gradually risen from 96° to 98°. As the washings from the abdominal cavity have returned perfectly clear for two days past, I removed the cannula to-day. Patient calls my attention to a swelling of the left parotid gland.

31st. The glandular swelling has been extreme, but is now subsiding.

Jan. 3d. The glandular swelling has disappeared. Patient much better. She sat up in bed to-day, the first time since the operation, and sang.

8th. The abdominal wound has been inflamed and discharged pus for the past ten days, and to-day the pedicle ligature escaped from the sinus in its lower portion.

Feb. 11th. To-day the patient sat up in a chair. She seemed quite strong. Some pus still discharges through the opening in the vaginal wall left by the cannula.

27th. Is walking about her room, and seems quite convalescent.

March 3d. Another set-back. Since early this morning the left leg and foot have been rapidly swelling, and the pulse has risen from 100 to 128. The limb is not very painful; it pits on pressure, and is tender along the course of the femoral vein. The urine is turbid and scanty. She has, clearly, phlegmasia dolens.

20th. The swelling has subsided. Patient's appetite has returned, and the abdominal wound has entirely healed.

May 15th. She now does light work about the house, takes long drives into the country, and has gained fifteen pounds in weight.

*Remarks.*—There are several noteworthy features connected with this case. One is the occurrence of very decided symptoms of septic poisoning, and their prompt relief on the removal of the pelvic collection of decomposed bloody fluid; showing clearly the necessity, in all cases in which there is reason to apprehend any effusion of blood after ovariotomy, of providing at the time for efficient drainage; or, if this be not done, of promptly reaching the interior of the abdominal cavity either by vaginal puncture or reopening of the abdominal incision, for the double purpose of evacuating septic material and of subsequent intra-peritoneal washings. There can be no doubt but that this patient would have died had the accumulation of fluid in the pelvis been permitted to remain. The rubber tubing employed in this case, although it had a calibre of $\frac{1}{4}$ inch, and was pierced with numerous openings $\frac{1}{32}$ inch in diameter was quite inefficient for its intended purpose. Its fenestra soon became clogged, and the small amount of fluid which was transmitted by it gave an erroneous impression as to the actual condition of the parts within. Another objection to rubber in these cases is, that unless the tubing be of large size—necessitating the use of a correspondingly large trocar for its introduction—its walls are likely to be compressed at the point where it passes through the vagina. A glass or metal tube, properly curved, reaching from the middle of Douglas' space to half way through the vagina, would, I think, be found much more efficient and manageable. It could be prevented from passing into the abdominal cavity by a tightly-fitting collar of india rubber slipped upon it, and, if desirable, a piece of tubing could be attached to its distal extremity. The tube itself should not project beyond the vulva, because of its liability to be caught in the bedding, and thus, or in other manner, pulled from its place.

Again, the case is remarkable for the very unusual character of the complications attending convalescence. These were a distinct attack of unilateral mumps, and a well developed phlegmasia dolens.

Finally, the temperature of the patient throughout was notably low. An hour after the operation it was 98°, and it remained without variation at this point during the first six days. It then fell to 94°, and, on the following day rose to 96°; keeping thus for nine days. It rose on the sixteenth day to 98°, simultaneously with the attack of parotitis, but at no time did it rise above a normal point.

